# *Saccharomyces boulardii* Mitigates Fructose-Induced Non-Alcoholic Fatty Liver in Rats

**DOI:** 10.3390/medicina60101713

**Published:** 2024-10-18

**Authors:** Mehmet Ulusan, Mumin Alper Erdogan, Ozkan Simsek, Vehbi Gunes, Oytun Erbas

**Affiliations:** 1 Department of Internal Medicine, Faculty of Veterinary Medicine, Erciyes University, Kayseri 38280, Turkey; mehmet.bucak@hotmail.com (M.U.); vgunes@erciyes.edu.tr (V.G.); 2 Department of Internal Medicine, Faculty of Veterinary Medicine, Burdur Mehmet Akif Ersoy University, Burdur 15030, Turkey; 3 Department of Physiology, Faculty of Medicine, Izmir Katip Celebi University, Izmir 35620, Turkey; alpero86@gmail.com; 4 Department of Physiology, Faculty of Veterinary Medicine, Burdur Mehmet Akif Ersoy University, Burdur 15030, Turkey; 5 Experimental Research and Application Center (DEKAM), Erciyes University, Kayseri 38280, Turkey; 6 Department of Physiology, Faculty of Medicine, Istanbul Demiroglu Bilim University, Istanbul 34394, Turkey; oytunerbas@yahoo.com

**Keywords:** fatty liver, fructose, saccharomyces boulardii, aquaporin-8, sirtuin-1, oxidative stress

## Abstract

*Background and Objectives:* Non-alcoholic fatty liver disease (NAFLD) is a growing global health concern closely linked to metabolic disorders, including obesity, insulin resistance, and dyslipidemia. Emerging evidence suggests that the gut–liver axis plays a critical role in the pathogenesis of NAFLD, with recent research highlighting the influence of gut microbiota, including fungal species such as Saccharomyces boulardii (*S. boulardii*). This study aimed to evaluate the effects of *S. boulardii* on lipid metabolism and oxidative stress in a rat model of fructose-induced NAFLD. *Materials and Methods:* Thirty Wistar rats were divided into three groups: a control group, a fatty liver group induced by 35% fructose supplementation, and a treatment group receiving *S. boulardii* (100 mg/kg/day) after fructose induction. *Results:* Biochemical analyses revealed that the treatment group exhibited significantly lower plasma levels of malondialdehyde (MDA), alanine aminotransferase (ALT), total triglycerides, and cholesterol compared to the untreated fatty liver group (*p* < 0.05). Furthermore, liver tissue analysis showed a marked reduction in lipid accumulation and fatty infiltration in the treatment group, with no visible lipid vacuoles in hepatocytes. The expression of aquaporin-8 (AQP8) and sirtuin-1 (SIRT1), key markers associated with hepatocyte function and lipid metabolism, was significantly higher in the *S. boulardii* group compared to the fatty liver group (*p* < 0.001). *Conclusions:* These findings indicate that *S. boulardii* supplementation mitigates the metabolic and oxidative stress-related alterations associated with fructose-induced NAFLD. In conclusion, our study suggests that *S. boulardii* exerts protective effects on the liver by reducing lipid accumulation and oxidative stress, highlighting its potential as a therapeutic intervention for NAFLD.

## 1. Introduction

Non-alcoholic fatty liver disease (NAFLD) is a disorder characterized by the accumulation of hepatic fat in patients with no excessive alcohol consumption. The development of NAFLD is primarily associated with metabolic disturbances, including insulin resistance, obesity, type 2 diabetes, and lipid metabolism disorders [1,2,3,4]. NAFLD is one of the most common causes of liver disease globally. The incidence of NAFLD, which is associated with a high mortality rate, is increasing and ranges from 24 to 45% worldwide [5].

Numerous studies have established a correlation between NAFLD and elevated levels of specific biochemical markers, including alanine aminotransferase (ALT), triglycerides, total cholesterol, and indicators of hepatic lipid accumulation and damage [6,7,8,9]. Conversely, a reduction in certain proteins, such as AQP8 and SIRT1, has been observed in animal models of NAFLD [10,11]. Aquaporin-8 is a transmembrane protein that facilitates water transport within hepatocytes [12]. Sirtuin-1 serves as a pivotal regulatory protein in hepatic lipid metabolism, oxidative stress, and inflammation, functioning through the deacetylation of transcriptional regulators. These proteins are adversely affected in NAFLD [10,11].

Previous studies have indicated that the gut microbiota is altered in terms of the diversity and abundance of bacteria and fungi in various liver diseases, including alcohol-associated liver disease [13], obesity [14,15], cirrhosis [16], primary sclerosis cholangitis [17], hepatocellular carcinoma [18], and NAFLD [2,5,19,20]. The connection between the liver and gut microbiota is bidirectional, mediated via the biliary and portal venous systems, and is known as the gut–liver axis. Therefore, the effect of bacteria that form a part of the intestinal microbiota has been extensively investigated in studies on liver diseases [5,14,20,21,22,23]. Recently, there has been an increase in studies focusing on fungi in the gut microbiota, as they have been recognized as fundamental components [5,22].

Saccharomyces boulardii is a fungus that has been studied for its effects on liver disease due to its probiotic properties. Research has shown that it can alter the gut microbiome and alleviate hepatic steatosis, acute liver injury, and low-grade inflammation [5,23,24]. When this probiotic was administered to animals fed a high-fat diet for 8 weeks, it was observed to reduce body weight, fatty liver, and inflammation levels in the rats [5,25].

Oxidative stress and lipid peroxidation are significant pathophysiological pathways that increase in NAFLD. The concentration of MDA in the liver can be measured to detect oxidative stress [26,27,28]. Previous studies have found that probiotics can offer protection against oxidative stress by reducing reactive oxygen species [20,29,30]. Saccharomyces boulardii, known for its probiotic properties, also exhibits potent antioxidant activity [31,32,33]. Barssotti et al. [33] demonstrated that *S. boulardii* effectively reduced oxidative stress in mice with diabetes.

In light of this information, our study had three objectives: (i) to investigate the effect of *S. boulardii* supplementation on liver biochemical parameters; (ii) to assess the effect of *S. boulardii* on lipid accumulation; and (iii) to evaluate the impact of *S. boulardii* on lipid peroxidation in rats with fructose-induced fatty liver.

## 2. Materials and Methods

### 2.1. Animals

Thirty adult male Wistar rats, each weighing between 200 and 210 g, were used for this study. The animals were housed under standard conditions and maintained on a 12 h light/dark cycle at a temperature of 22 ± 2 °C. Throughout the experiment, the rats were given free access to a standard pellet diet (35% fat, 18% protein, and 47% carbohydrates) and tap water. The experimental protocol received approval from the Institutional Animal Care and Ethical Committee of the University of Science University (protocol code: 0123123109). The maintenance of animals was conducted in accordance with the standards set in the International Guide for the Care and Use of Laboratory Animals.

### 2.2. Experimental Protocol

The abovementioned 30 Wistar rats were randomly divided into three experimental groups, each consisting of ten animals: the control group, the high-fructose group, and the treatment group. The control group was fed a standard chow diet. In contrast, the high-fructose and treatment groups received 35% fructose added to their drinking water for 8 weeks to induce hepatosteatosis as described previously [34]. After this induction phase, the animals in the high-fructose group were administered 1 mL/kg/day of tap water, while the treatment group received 100 mg/kg/day of *S. boulardii* (Florastor, Biocodex, Gentilly, France) via gavage for a period of 4 weeks.

At the conclusion of the experiment, all rats were euthanized through cervical dislocation under anesthesia using a combination of 100 mg/kg ketamine and 10 mg/kg xylazine [35]. Blood samples were then collected via cardiac puncture for biochemical analysis, and liver tissues were harvested for both histopathological and biochemical evaluations. All reagents were obtained from Sigma-Aldrich Inc., unless specified otherwise.

### 2.3. Plasma Lipid Peroxidation Analysis

Lipid peroxidation in the plasma was assessed by measuring malondialdehyde levels, which were determined as thiobarbituric acid reactive substances (TBARSs). The procedure involved adding trichloroacetic acid and TBARS reagent to the plasma samples, followed by thorough mixing. The samples were then incubated at 100 °C for 60 min. After cooling on ice, the samples were centrifuged for 20 min at 3000 rpm. The absorbance of the supernatant was then measured at 535 nm. MDA concentrations were expressed in nM, and tetraethoxypropane was used as the calibration standard [36].

### 2.4. Blood Biochemistry

Blood samples were collected via ventricular puncture using a 1 mL syringe and transferred into heparinized tubes. To separate the plasma, the samples were centrifuged at 3000 rpm for 10 min at 22 ± 2 °C. The resulting plasma was then stored at −20 °C until further analysis. Plasma levels of alanine ALT, triglycerides, and total cholesterol were measured across all groups using a Beckman-Coulter AU 640 autoanalyzer along with commercially available kits (Beckman Coulter Inc., Brea, CA, USA).

### 2.5. Liver Biochemical Analysis

Following euthanasia, the liver was promptly removed and stored at −20 °C for subsequent biochemical evaluation. The liver tissue was homogenized in 5 volumes of phosphate-buffered saline (PBS) at pH 7.4 using a glass homogenizer and then centrifuged at 5000 rpm for 15 min. The supernatant was collected for analysis of total protein content using the Bradford method with bovine serum albumin as the calibration standard [37].

The concentrations of AQP8 and SIRT1 in the liver homogenates were measured using enzyme-linked immunosorbent assay (ELISA) kits provided by a commercial supplier. The samples were processed in duplicate according to the manufacturer’s instructions. Absorbance readings were recorded using a MultiscanGo microplate reader (Thermo Fisher Scientific Inc., Portsmouth, NH, USA).

### 2.6. Histopathological Assessment of the Liver

For histological and immunohistochemical analyses, the rats were anesthetized with an intraperitoneal injection of ketamine (100 mg/kg) and xylazine (10 mg/kg), followed by perfusion with 200 mL of 4% formaldehyde in 0.1 M PBS. Formalin-fixed liver tissues, sectioned at 4 μm thickness, were stained with hematoxylin–eosin for examination. All tissue samples were imaged using an Olympus C-5050 digital camera (Olympus Co., Tokyo, Japan) mounted on an Olympus BX51 microscope (Olympus Co., Tokyo, Japan).

For the morphological analysis, a computerized image analysis system (Image-Pro Express 1.4.5, Media Cybernetics, Inc., Rockville, MD, USA) was employed. Ten microscopic fields per liver section were assessed at 40× magnification by an observer who was blinded to the experimental groups. The percentage of fatty infiltration in the liver tissues from each group was evaluated.

### 2.7. Statistical Analysis

Statistical analysis was performed using SPSS version 15.0. Student’s *t*-test and analysis of variance (ANOVA) were used to analyze parametric variables, while the Mann–Whitney U test was employed for non-parametric data. The Shapiro–Wilk test was also applied to assess both parametric and non-parametric variables. All results are expressed as mean ± standard error of the mean (SEM). A *p*-value of less than 0.05 was considered statistically significant.

## 3. Results

### 3.1. Effects of S. boulardii on Body Weight, Food Consumption, and Tissue Weights

Body weights were recorded weekly, and food consumption was monitored daily. No significant differences in food intake were observed between the control (25.4 g/day), high-fructose (25.6 g/day), and treatment groups (25.5 g/day), suggesting that differences in body weight were not attributable to food consumption. The high-fructose group exhibited a significant increase in final body weight (300.4 g) compared to the control group (250.3 g, *p* < 0.05). In contrast, the treatment group had a significantly lower final body weight (260.1 g, *p* < 0.05), indicating that *S. boulardii* supplementation mitigated the fructose-induced weight gain (Table 1).

Liver and adipose tissue weights were recorded at the end of the experiment. The high-fructose group had significantly higher liver weights (12.5 g) and adipose tissue weights (10.8 g) compared to the control group (liver: 8.2 g; adipose: 6.4 g; *p* < 0.05). The treatment group, however, showed a marked reduction in liver (9.1 g) and adipose tissue weights (7.0 g; *p* < 0.05), indicating that *S. boulardii* supplementation prevented excessive fat accumulation and liver enlargement associated with fructose-induced NAFLD (Table 1).

### 3.2. Histopathological and Quantitative Analyses of Lipid Accumulation

**Figure 1:**
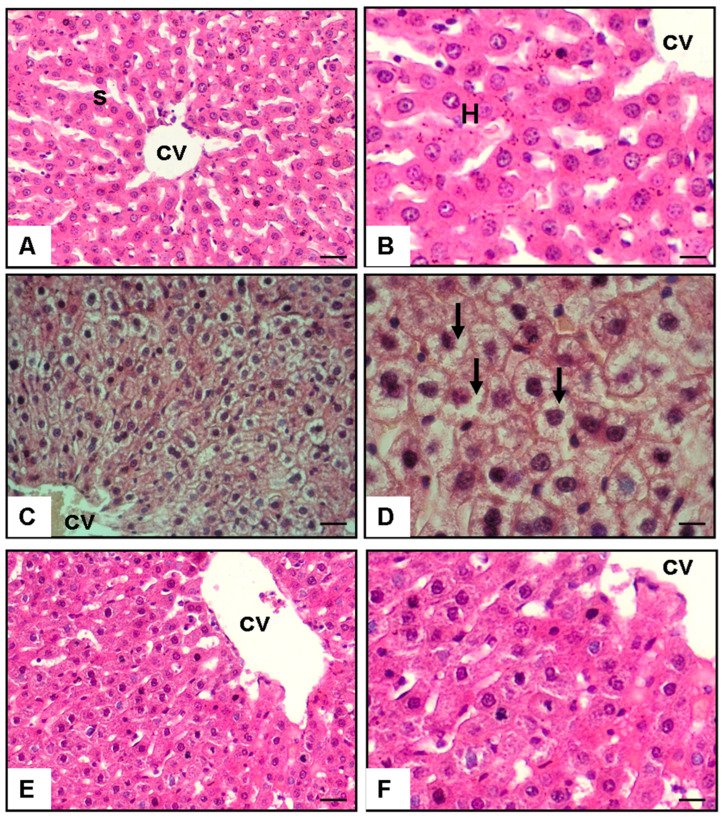
Effect of *S. boulardii* on lipid accumulation in hepatocytes: histological and quantitative analyses. Hematoxylin–eosin staining of sections from rat liver at magnification of 40× and 100×. (**A**,**B**) Control group; S: sinusoid; H: hepatocyte; CV: central vein; (**C**,**D**) 35% fructose and tap water group liver; arrow indicates lipid accumulation in the hepatocytes as vacuoles; (**E**,**F**) 35% fructose and 100 mg/kg/day *S. boulardii* group.

Hematoxylin–eosin-stained sections of rat liver tissue, viewed at 40× and 100× magnification, are shown in Figure 1. The findings revealed that hepatocytes in the high-fructose group exhibited significant lipid accumulation in the form of large vacuoles, a feature absent in the control group. In the treatment group, which received *S. boulardii* at a dose of 100 mg/kg/day, there was a marked reduction in lipid accumulation within the hepatocytes compared to the high-fructose group. Although small lipid vacuoles were still present, they were considerably fewer and smaller than those observed in the high-fructose group. This suggests that *S. boulardii* significantly mitigated, but did not completely eliminate, lipid infiltration in the liver (Figure 1).

Quantitative analysis of lipid accumulation in hepatocytes, measured as the percentage of hepatocytes containing lipid vacuoles, showed significant differences among the groups. The high-fructose group exhibited a markedly higher percentage of hepatocytes with lipid vacuoles (85.4%) compared to the control group (1.3%, *p* < 0.001), indicating extensive lipid infiltration as a result of high fructose-induced NAFLD. In contrast, the treatment group, which received S. boulardii, demonstrated a significant reduction in lipid accumulation, with only 11.8% of hepatocytes showing lipid vacuoles (*p* < 0.001 vs. high-fructose group). Although this reduction was substantial, the treatment group still had a higher percentage of lipid vacuoles compared to the control group (*p* < 0.05), indicating that *S. boulardii* mitigated but did not completely eliminate lipid infiltration in hepatocytes (Figure 1).

### 3.3. Effects of S. boulardii on Biochemical Parameters

The levels of MDA, ALT, total triglycerides, total cholesterol, AQP8, SIRT1, and fatty infiltration in both plasma and tissue samples of the rats are presented in Table 2. The results demonstrated that plasma concentrations of ALT (U/L), MDA (nmol/mg protein), triglycerides (mg/dL), total cholesterol (mg/dL), and the percentage of fatty infiltration were significantly elevated in the high-fructose group compared to the control group (*p* < 0.05). However, in the treatment group receiving 100 mg/kg/day *S. boulardii*, these parameters were notably reduced compared to the high-fructose group. Additionally, AQP8 (pg/mg protein) and SIRT1 (pg/g protein) levels were significantly lower in the high-fructose group than in the control group, whereas these markers showed increased levels in the treatment group compared to the high-fructose group (Table 2).

## 4. Discussion

Non-alcoholic fatty liver disease is a growing global health issue, which can become life-threatening in its advanced stages [38]. Numerous studies have highlighted a strong association between gut microbiota and the development of liver damage [20,22,23,24,39,40]. Many studies have also examined the role of probiotics in liver disease. Research on *S. boulardii*, a fungal probiotic, has shown its ability to alleviate hepatic steatosis, acute liver injury, low-grade inflammation, and promote antioxidant activity by modulating the gut microbiota [5,22,23,24]. Based on these findings, our study explored the gut–liver axis and the biochemical and histopathological effects of *S. boulardii* supplementation in a rat model of NAFLD.

Previous research has indicated that parameters such as ALT, triglycerides, total cholesterol, and fatty infiltration hallmarks of NAFLD are elevated due to lipid accumulation and liver damage [6,7,8,9]. In line with this, our study also found elevated levels of these markers in the fructose-induced fatty liver group. However, administration of *S. boulardii* in this group resulted in reductions in plasma ALT, triglycerides, total cholesterol, and fatty infiltration. This suggests that *S. boulardii* reduced fat accumulation in the liver, thereby mitigating the damage.

In both clinical and in vivo studies, elevated lipid peroxidation has been observed in cases of fatty liver disease [28,41,42]. Disrupted lipid metabolism leads to fat accumulation in the liver, increasing MDA levels due to the activation of reactive oxygen species producers such as the mitochondria and endoplasmic reticulum [43,44,45]. In agreement with these findings, our study found that MDA levels were higher in the fructose-induced fatty liver group compared to controls. However, the administration of *S. boulardii* led to a significant reduction in MDA levels, likely due to the probiotic’s antioxidant properties, as reported in previous studies [31,32].

Probiotics have been demonstrated to exert a protective effect on liver cells through a number of different mechanisms. Primarily, it enhances the intestinal barrier function by regulating the intestinal microbiota, thereby reducing the amount of toxins reaching the liver as a consequence of harmful bacteria. Secondly, due to their anti-inflammatory effects, they produce substances that reduce inflammation. These substances prevent cell damage by reducing the inflammatory response in the liver. Furthermore, they produce antioxidants that neutralize free radicals, thereby exerting a protective effect on hepatocytes. Finally, they prevent liver cell damage by preventing excessive immune responses due to immune system modulation [46,47]. The present study demonstrated that *S. boulardii* was effective in preventing liver damage due to its beneficial impact on oxidative stress parameters, subsequently improving non-alcoholic fatty liver disease.

In addition to analyzing lipid parameters and oxidative stress markers, we assessed the expression levels of AQP8 and SIRT1 in liver tissue. Both AQP8 and SIRT1 have been shown to play critical roles in hepatic function, particularly in lipid metabolism, oxidative stress, and inflammation, processes that are central to the pathogenesis of NAFLD. Aquaporin-8 is a transmembrane water channel protein involved in maintaining water homeostasis in hepatocytes and facilitating bile secretion. Previous studies have demonstrated that AQP8 expression is downregulated in liver diseases such as cholestasis, hepatic steatosis, and NAFLD, leading to hepatocyte swelling and lipid accumulation due to impaired water transport [10,11,12]. Consistent with this, our results showed a significant decrease in AQP8 levels in the fatty liver group compared to the control group. However, treatment with *S. boulardii* significantly restored AQP8 expression, suggesting that the probiotic may play a role in maintaining water balance in hepatocytes and alleviating the pathological effects of lipid accumulation.

SIRT1, a key regulator of hepatic lipid metabolism, oxidative stress, and inflammation, has been linked to the pathogenesis of NAFLD. Reduced SIRT1 activity has been associated with increased hepatic steatosis, insulin resistance, and liver inflammation [48,49]. Our study found a marked reduction in SIRT1 levels in the fatty liver group, which was consistent with previous findings that SIRT1 is downregulated in NAFLD [49]. In contrast, the administration of *S. boulardii* significantly upregulated SIRT1 expression, suggesting that the probiotic may contribute to improved lipid metabolism and reduction in oxidative stress and inflammation, ultimately protecting against the progression of NAFLD.

Histopathological analysis revealed that, in the treatment group that received *S. boulardii*, a significant reduction in lipid accumulation was observed compared to that in the high-fructose group. Although some lipid vacuoles were present, they were substantially fewer and smaller in size than those seen in the fatty liver group, where hepatocytes exhibited extensive lipid accumulation. This suggests that *S. boulardii* effectively mitigated but did not completely eliminate lipid accumulation. These findings align with the existing literature on the role of probiotics in improving lipid metabolism and reducing hepatic steatosis [5,24,50,51].

## 5. Conclusions

In conclusion, this study demonstrated the biochemical and histopathological benefits of oral *S. boulardii* supplementation in a rat model of fatty liver disease. Our results showed that *S. boulardii* reduced plasma ALT, triglycerides, total cholesterol, fatty infiltration, and MDA levels while increasing AQP8 and SIRT1 in the liver. Additionally, histological analysis confirmed a reduction in hepatic lipid accumulation in *S. boulardii*-treated rats. These findings suggest that this fungal probiotic may serve as a potential adjunct therapy for NAFLD, contributing to the reduction in hepatic fat and having beneficial effect on key molecular pathways involved in water transport and lipid metabolism.

## Figures and Tables

**Table 1:** Phenotypic data of the control, high-fructose, and treatment groups.

	Initial Body Weight (g)	Final Body Weight (g)	Food Consumption (g/day)	Liver Weight (g)	Adipose Tissue Weight (g)
**Control Group**	200.2 ± 2.1	250.3 ± 5.4	25.4 ± 0.3	8.2 ± 0.5	6.4 ± 0.4
**High-Fructose Group**	199.8 ± 2.3	300.4 ± 6.2	25.6 ± 0.4	12.5 ± 1.0	10.8 ± 0.8
**Treatment Group**	200.5 ± 2.0	260.1 ± 4.9	25.5 ± 0.3	9.1 ± 0.6	7.0 ± 0.5

Data are expressed as mean ± SEM. Statistical significance was assessed using one-way ANOVA followed by post hoc Tukey’s test. *p* < 0.05: high-fructose group vs. control group for final body weight, liver weight, and adipose tissue weight. *p* < 0.05: treatment group vs. high-fructose group for final body weight, liver weight, and adipose tissue weight. There were no significant differences in food consumption between the groups. Control Group; Standard chow diet; High-Fructose Group; 35% fructose and 1 mL/kg/day tap water; Treatment Group; 35% fructose and 100 mg/kg/day *S. boulardii*.

**Table 2:** Effects of *S. boulardii* on some biochemical parameters in rats with fructose-induced fatty liver.

	Control Group	High-Fructose Group	Treatment Group
**Aminotransferase (U/L)**	41.7 ± 2.06	59.4 ± 4.6 *	45.9 ± 3.2 ^#^
**Plasma Malondialdehyde Level (nmol/mg protein)**	32.8 ± 1.2	51.7 ± 2.2 *	33.5 ± 1.7 ^#^
**Plasma Triglyceride (mg/dL)**	32.3 ± 1.9	56.4 ± 5.1 *	43.4 ± 1.8 ^#^
**Plasma Total Cholesterol (mg/dL)**	50.5 ± 7.1	126.8 ± 9.3 **	101.3 ± 6.07 ^#^
**Liver Aquaporin-8 Level** **(pg/mg protein)**	3.6 ± 0.1	2.4 ± 0.1 *	3.5 ± 0.2 ^##^
**Liver Sirtuin-1 Level (pg/g protein)**	4.06 ± 0.3	3.1 ± 0.08 *	3.8 ± 0.09 ^#^
**Fatty Infiltration Cell (percent)**	1.3 ± 0.2	85.4 ± 5.7 **	11.8 ± 3.5 ^##^

Data are shown as mean ± SEM. * *p* < 0.05, ** *p* < 0.001 (difference from the control group), ^#^ *p* < 0.05, ^##^ *p* < 0.001 (difference from the high-fructose group). Control Group; Standard chow diet; High-Fructose Group; 35% fructose and 1 mL/kg/day tap water; Treatment Group; 35% fructose and 100 mg/kg/day *S. boulardii.*

## Data Availability

All data obtained from this study are included in this article.
